# Intracellular transport dynamics revealed by single-particle tracking

**DOI:** 10.52601/bpr.2021.210035

**Published:** 2021-10-31

**Authors:** Ming-Li Zhang, Hui-Ying Ti, Peng-Ye Wang, Hui Li

**Affiliations:** 1 School of Systems Science, Beijing Normal University, Beijing 100875, China; 2 Beijing National Laboratory for Condensed Matter Physics and Laboratory of Soft Matter Physics, Institute of Physics, Chinese Academy of Sciences, Beijing 100190, China; 3 School of Physical Sciences, University of Chinese Academy of Sciences, Beijing 100049, China; 4 Songshan Lake Materials Laboratory, Dongguan 523808, Guangdong , China

**Keywords:** Intracellular transport, Single-particle tracking (SPT), Diffusive motion, Directed motion, Near infrared SPT, Dynamics

## Abstract

Intracellular transport is the basis for the transfer of matter, energy, and information in cells and is critical to many cellular functions. Within the nonequilibrium environment of living cells, the transport behaviours are far from the traditional motion in liquid but are more complex and active. With the advantage of high spatial and temporal resolution, the single-particle tracking (SPT) method is widely utilized and has achieved great advances in revealing intracellular transport dynamics. This review describes intracellular transport from a physical perspective and classifies it into two modes: diffusive motion and directed motion. The biological functions and physical mechanisms for these two transport modes are introduced. Next, we review the principle of SPT and its advances in two aspects of intracellular transport. Finally, we discuss the prospect of near infrared SPT in exploring the *in vivo* intracellular transport dynamics.

## INTRODUCTION

Intracellular transport dynamics are critical to many cellular functions, such as cell proliferation, motility, and death (Mogre* et al.* 2020). Almost all biochemical reactions in the cell rely on the intracellular transport of biomolecules. Proteins synthesized in ribosomes usually undergo transportation from the perinuclear regions to the peripheral sites in the cell or are secreted outside the cell membrane (Schwarz and Blower 2016). As the energy unit in cells, ATP is produced in the mitochondria and transported to different sites within the cell for consumption. External signals received by the cell membrane usually need to be transmitted to the nucleus to initiate transcriptional responses. In short, intracellular transport is the basis for the transfer of matter, energy, and information.

**Figure 1:**
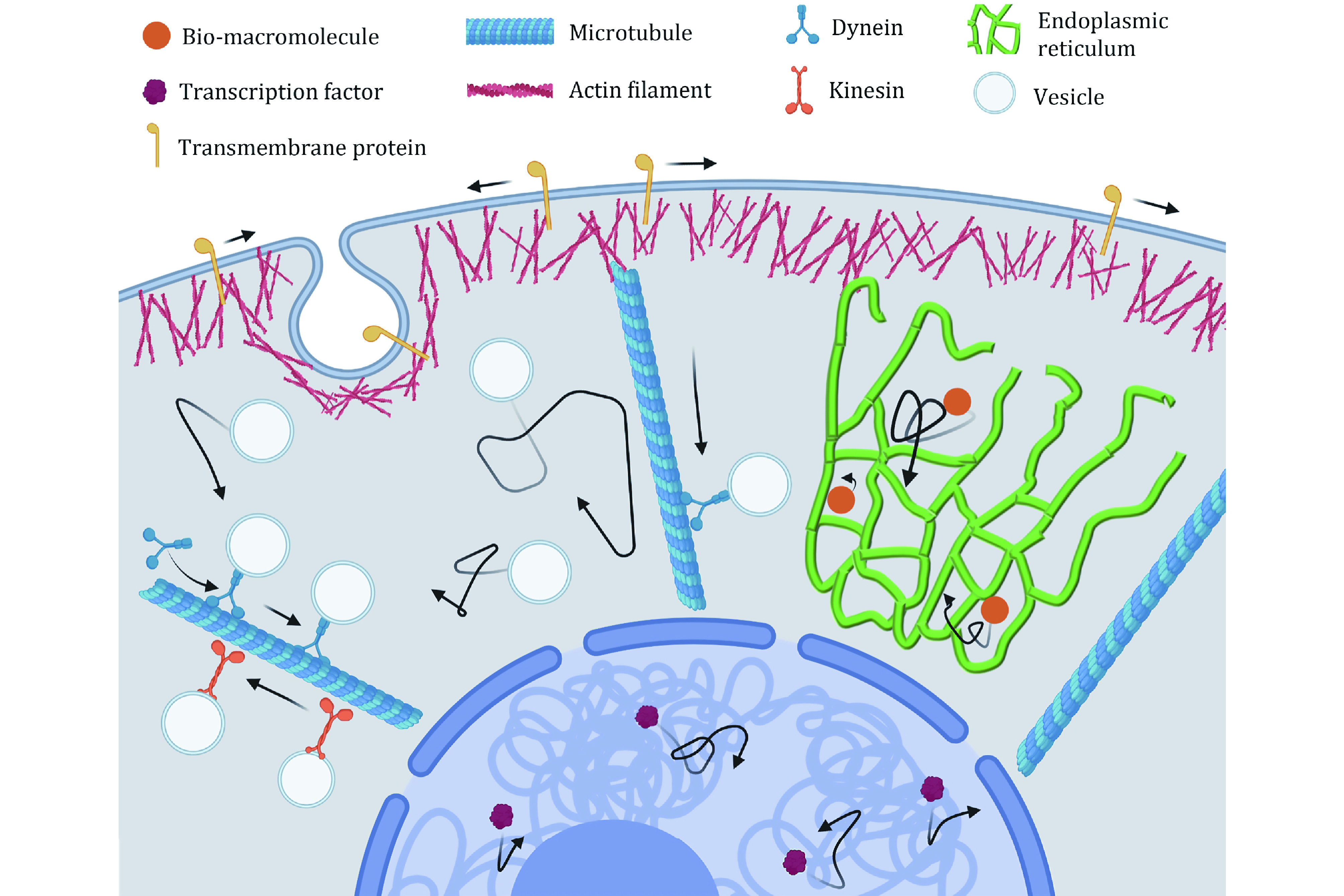
Schematic of intracellular transport. In cytoplasm, the diffusion of vesicles and bio-macromolecules, and the directed motion of vesicles driven by motor proteins along microtubules. In nucleus, the diffusion of the transcription factors

The living cell is a nonequilibrium system in which many energy-consuming reactions give rise to complex and active transport behaviours (Gallet* et al.* 2009; Wilhelm 2008). In addition, macromolecule crowding and spatial heterogeneity have significant impacts on intracellular transport. As such, molecule transport dynamics in living cells is far from traditional motion in liquid. According to the motion type, transport in eukaryotic cells can be divided into random diffusion and directed transport from a physical perspective (Fig. 1).

Exploring the dynamics of intracellular transport requires advanced technologies. In recent decades, the development of noninvasive techniques for visualizing dynamic processes in living cells has greatly promoted our understanding of intracellular transport, including fluorescence recovery after photobleaching (FRAP) (Lippincott-Schwartz* et al.* 2003; Reits and Neefjes 2001) and fluorescence correlation spectroscopy (FCS) (Bulseco and Wolf 2007; Elson 2011; Haustein and Schwille 2007; Kim* et al.* 2007; Tudor* et al.* 2007; Vukojevic* et al.* 2007). However, the above techniques provide ensemble average results, lacking the spatiotemporal dynamics of individual molecules. Single-particle tracking (SPT) enables us to visualize individual molecules in living cells, locate single molecules with nanoscale precision, and measure their individual transport dynamics as a function of time. In terms of the spatiotemporal information from single molecule trajectories, we can further probe the heterogeneous environment within the cell. Many advances have been made in the study of intracellular transport dynamics through SPT (Balint* et al.* 2013; Cognet* et al.* 2014; Ge* et al.* 2021; Hui* et al.* 2017; Jiang* et al.* 2020; Kusumi* et al.* 2014; Li* et al.* 2015b, 2016b, 2018a; von Diezmann* et al.* 2017; Xu* et al.* 2021).

This review is organized as follows. First, we introduce the biological functions of intracellular transport. Next, we describe two types of intracellular transport and their physical mechanisms. Then, we concentrate on the fundamental principle of SPT technology, including imaging and data analysis. After that, we focus on the applications of SPT in intracellular studies. Finally, we discuss future studies of intracellular transport dynamics, especially strategies for developing *in vivo* SPT methods.

## BIOLOGICAL MEANING OF INTRACELLULAR TRANSPORT

### Matter translocation

Matter translocation plays an important role in maintaining the physiological functions of cells. The secretions synthesized by cells are transported from intracellular to extracellular space. For example, synthesized proteins are translocated from perinuclear to peripheral sites or eventually secreted outside the cell membrane. In eukaryotes, proteins are first synthesized in ribosomes attached to the rough endoplasmic reticulum (ER) (Schwarz and Blower 2016). Then, after further modifications in the Golgi (Orci* et al.* 2000), proteins are transported in vesicles to the plasma membrane or secreted into the extracellular matrix. Conversely, extracellular biomolecules that enter the membrane undergo intracellular transport in the cytoplasm. For example, extracellular small molecules such as amino acids cross the cell membrane by diffusion, while macromolecules such as growth factors, membrane proteins, and lipids pass through the cell membrane via endocytosis and are then translocated within the cell (Basturea 2019). Viruses, such as influenza, enter the cell through endocytosis and undergo intracellular transport to their destinations (Lakadamyali* et al.* 2003).

In cells, diffusion is the major mechanism for matter translocations. However, the diffusion coefficients of large cargos such as the vesicles in oocytes and lysosomes in epithelial cells are only 0.003 and 0.071 μm^2^/s, respectively (Bandyopadhyay* et al.* 2014; Drechsler* et al.* 2017; Koslover* et al.* 2016). At such slow diffusion rates, it would take several hours for the large cargos to cross the cell. To be more efficient, cells utilize motor-driven active transport, which is essential in eukaryotic cells (De Matteis and Luini 2008). For example, in neurons which can be up over 1-metre long, axon cargos are transported along microtubules by motor proteins, including kinesin and dynein (Mudrakola* et al.* 2009).

### Energy transfer

Energy is stored in the form of ATP or nutrients such as carbohydrates and fats, playing indispensable roles in intracellular metabolism and various life activities. Small molecules such as ATP and glucose are rapidly transported within the cell by diffusion, with diffusion rates of approximately 145 and 200 μm^2^/s, respectively (Riley* et al.* 1999; Vendelin* et al.* 2000). Due to the low efficiency of diffusion in large-scale transport, actively directed transport prevails in large cells. For example, in individual hyphae, nutrients such as N-rich amino acids are translocated by both diffusion and vesicle transport; however, in the mycelial network, vesicle transport dominates (Fricker* et al.* 2017, 2008).

### Signal transduction

When the cell membrane senses and receives signals from the extracellular environment, the signals in the form of active small molecules are usually transmitted to the nucleus, in which certain transcriptional regulation takes place to trigger cellular responses. Intracellular signal transduction involves the diffusion of activated proteins from the cell membrane to cytoplasmic targets or other cellular sites. For example, MEK kinase is activated at the membrane and then diffuses into the cytoplasm to activate downstream kinases (Kholodenko* et al.* 2000). In addition, vesicle transport driven by motor proteins is an efficient alternative. A typical example is the internalization and endocytic trafficking of nerve growth factors in neuronal signalling pathways (Harrington and Ginty 2013). Besides, the cellular mechanotransduction as a growing area is also related to the intracellular transport (He* et al.* 2020; Liu* et al.* 2019; Wang* et al.* 2020).

## PHYSICAL MECHANISMS OF INTRACELLULAR TRANSPORT

To achieve various functional goals of intracellular transport, eukaryotic cells mainly rely on two mechanisms: random diffusion and directed transport. The diffusion is driven by the combination of thermal and intracellular active fluctuations, whereas the directed transport is driven by motor proteins along the cytoskeletons (Fig. 1). Both of them are influenced by the intracellular ATP levels and macromolecule crowding.

### Diffusion

The irregular motion of mesoscale particles suspended in a solvent is named Brownian motion and was first found by Robert Brown in 1826, who observed the continuously agitating motion of pollen grains under a light microscope (Brown 1828). Brownian motion is caused by the thermal fluctuations of water molecules. The diffusion coefficient, which represents the range of motion, increases with temperature. The time required for translocation by diffusion is proportional to the square of the distance. Moreover, the diffusion coefficient decreases with particle size or medium viscosity. Although diffusion is nondirectional, it works well for transporting small molecules over short distances (Di Rienzo* et al.* 2014).

However, intracellular diffusion is different from that observed in dilute solutions (Gregor* et al.* 2005; Jacobson and Wojcieszyn 1984; Lubyphelps* et al.* 1987). In some cases, the diffusive motion within the cell appears to be random, although the diffusion rate is much greater than the expected diffusion in solutions (Bursac* et al.* 2005; Lau* et al.* 2003). This suggests that other factors contribute to intracellular diffusion in addition to thermal fluctuations. The internal environment of living cells obviously deviates from the equilibrium state, in which many active processes consuming energy exist. Therefore, unlike thermal diffusion in an equilibrium system, the amplitude of diffusive motion in cells is additionally driven by active fluctuations, leading to randomly diffusive motions with increased diffusion rates (Brangwynne* et al.* 2009; Fakhri* et al.* 2014; Guo* et al.* 2014).

Individual molecule trajectories in living cells show nonlinear mean square displacement (MSD) as a function of time, which suggests anomalous diffusion (Saxton and Jacobson 1997; Wieser and Schutz 2008). This anomalous diffusion is attributed to the interactions between molecules and the surrounding intracellular environment, caused by the broad distribution of jump times or jump lengths, or the strong correlations (Bouchaud and Georges 1990). In most cases, the motion of macromolecules and organelles in the cytoplasm is subdiffusion (Hoffman* et al.* 2006; Li* et al.* 2016a; Shen* et al.* 2016; Tolic-Norrelykke* et al.* 2004; Yamada* et al.* 2000) due to viscoelastic properties and the crowded environment in the cytoplasm (Hofling and Franosch 2013; Luby-Phelps 2000; Shen* et al.* 2021; Wang* et al.* 2013; Weber* et al.* 2010).

In addition, the distribution of individual step sizes of small molecules in the cytoplasm shows a Laplace distribution, which is different from the desired Gaussian distribution in a uniform medium (Fodor* et al.* 2015; Gal* et al.* 2013; He* et al.* 2016; Lampo* et al.* 2017). The Laplace distribution is attributed to the wide distribution of diffusivities for individual particles in inhomogeneous environments (Luo and Yi 2018). Such a non-Gaussian distribution indicates the spatial heterogeneity of biomolecule motion in the cytoplasm (Chechkin* et al.* 2017; Duits* et al.* 2009). It should be noted that subdiffusion does not necessarily imply a non-Gaussian distribution. For example, fractional Brownian motion is anomalous diffusion but appears as a Gaussian distribution. In contrast, some normal diffusion can exhibit a non-Gaussian distribution (Chechkin* et al.* 2017; Wang* et al.* 2012).

### Directed motion

In the crowded environment of cells, the diffusion of large molecules and vesicles is physically constrained and is not sufficiently effective for long-distance transport. In this case, directed transport, which relies on motor proteins that hydrolyse ATP and drag the cargos along the cytoskeleton, is more applicable (Brown and Sivak 2020).

Cytoskeletons and molecular motors are essential for directed transport. Cytoskeletons, including microtubules and microfilaments (F-actin), are involved in intracellular transport. Microtubules, the structural backbone of the cytoskeleton, are long hollow tubes with a diameter of 22–25 nm composed of 13 parallel protofilaments. Microtubules are highly dynamic and polarized, alternating between phases of growth and shrinkage (de Forges* et al.* 2012). Microtubules provide the path for the long-range transport of organelles and membranes. Kinesin and dynein are motor proteins that transport along microtubules (Kikushima* et al.* 2013; Ross* et al.* 2008). In general, kinesins move towards the plus ends of microtubules from the perinucleus to the periphery (Duan* et al.* 2012; Hirokawa* et al.* 2009), whereas dynein drives retrograde movements towards the minus ends of microtubules from the periphery to the perinucleus (Cianfrocco* et al.* 2015; Reck-Peterson* et al.* 2018).

Another important cytoskeleton is the microfilament, which is a solid fibre with a diameter of 4–7 nm distributed beneath cell membranes and in the cytoplasm. Actin filaments are short and polarized. They usually form a randomly oriented network with a mesh size of approximately 50 nm (Barlan* et al.* 2013). Myosin motors mainly contribute to localized movements of cargo in a short range along actin filaments.

Directed motion has two major advantages. One is to transport intracellular cargo over relatively long distances. For example, in the axons of neurons, the distance can be up to one metre (Hirokawa* et al.* 2010). Another advantage is that the direction and speed transport dynamics can be well controlled by the cells (Burute and Kapitein 2019). For example, the ratio between kinesin and dynein can affect the direction of cargo transport, which has already been shown by *in vitro* experiments (Hendricks* et al.* 2010). A cargo can be driven by multiple motors which cooperate or compete with each other. When the same motors carry cargo, they may share the load and improve performance (Reis* et al.* 2012). When two different motor proteins attach to a cargo, it may be driven in two directions: if only one motor is active, it determines the cargo's direction of movement; if both motors are active, they will pull in opposite directions, with the stronger determining the direction of transport (Barlan* et al.* 2013; Gennerich* et al.* 2007; Hancock 2014; Hendricks* et al.* 2010; McLaughlin* et al.* 2016; Muller* et al.* 2008).

## PRINCIPLE OF SINGLE PARTICLE TRACKING

Advanced optical techniques have been applied to explore and understand transport dynamics within the intracellular world. One of the common techniques is fluorescence recovery after photobleaching (FRAP). In FRAP experiments, fluorescent molecules in a small given area are first photobleached by a focused laser beam with high intensity. Then, surrounding unbleached fluorescent molecules diffuse into the photobleached area resulting in fluorescence recovery (Lippincott-Schwartz* et al.* 2003; Reits and Neefjes 2001). The recovery of the fluorescence intensities is calculated to obtain the molecular diffusivity. Another technique is fluorescence correlation spectroscopy (FCS), which records the fluctuation of fluorescence intensity in a small, illuminated volume. The diffusion coefficient can be determined from the autocorrelation function of the fluorescence intensities (Bulseco and Wolf 2007; Elson 2011; Haustein and Schwille 2007; Kim* et al.* 2007; Peng* et al.* 2020; Tudor* et al.* 2007; Vukojevic* et al.* 2007).

In contrast to the above ensemble-averaged methods, SPT provides a new perspective of single molecule motion and a better understanding of intracellular transport dynamics. SPT provides spatiotemporal information about the movement of a single biomolecule, which helps to accurately measure different types of biomolecule transport in cells and understand its complexity. In SPT experiments, probes are used to label the molecules of interest, observe their movements under appropriate optical instruments, and analyse their trajectories to explore the intracellular transport dynamics.

### Probes

In experiments, the biomolecules first need to be labelled with proper probes, which enables observation and analysis of single-particle motion under microscopy. The probes must have good biocompatibilities with no harmful effects on cell activities. Moreover, the physical properties of the probes are of vital importance, and the probe cannot be so large that it impacts the original motion of biomolecules. To ensure that long trajectories can be followed, the probe should be photostable for long-term imaging.

**Figure 2:**
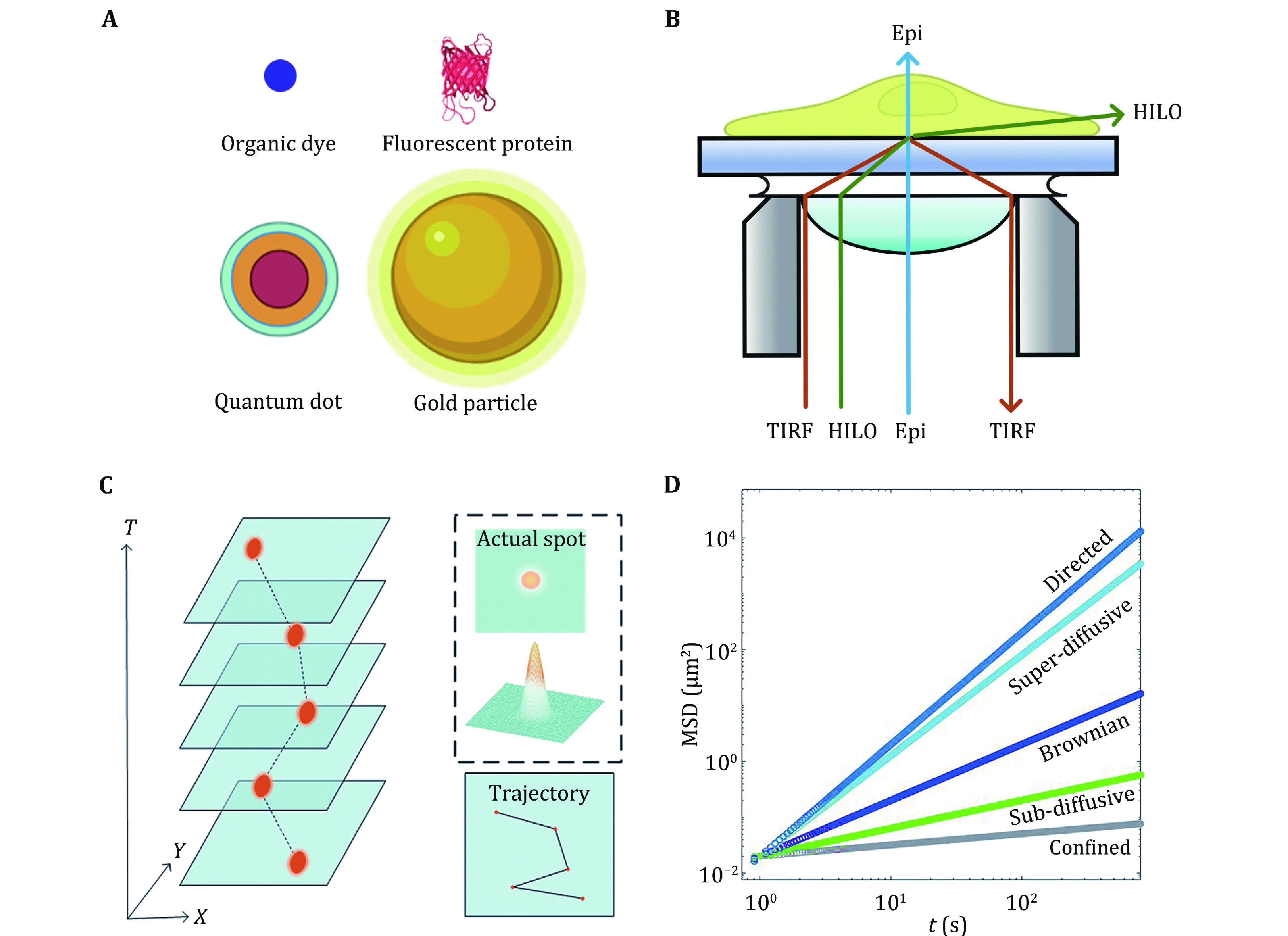
Principle of single particle tracking. **A** Commonly used probes. **B** Different illuminations in fluorescence microscopy. **C** Generation of trajectories. **D** MSD plots of different motion

Researchers have used gold particles (Kusumi* et al.* 1993), organic dyes (Schmidt* et al.* 1996), fluorescent proteins (Iino* et al.* 2001), and quantum dots (QDs) (Dahan* et al.* 2003) to label particles of interest (Fig. 2A). In 1993, Kusumi *et al*. labelled receptors on the cell membrane with 40-nm gold particles and observed the diffusive motion of the receptors (Kusumi* et al.* 1993). However, gold particles cannot be used in multiple-colour imaging.

In recent years, QDs have been considered ideal probes with excellent optical properties and biocompatibilities for *in vivo* and* in vitro* imaging (Dubertret* et al.* 2002; Larson* et al.* 2003; Zhou* et al.* 2015). In 2003, QDs were first used to label glycine receptors on the cell membrane (Dahan* et al.* 2003). QDs with narrow and symmetric emission spectra have strongly size-dependent emission wavelengths so that they can achieve multicolour SPTs (Zhou* et al.* 2015). Compared with other probes, QDs are quite photostable for long-term imaging. Although the application scope of QDs is very wide, their fluorescence intermittency (blinking) leads to incomplete molecular motion trajectories (Li* et al.* 2015c; Nirmal* et al.* 1996), bringing certain difficulties to data processing.

### Optical implementations

Fluorescence microscopy is indispensable to image probes and track their movements. In traditional epifluorescence microscopy (Epi), the laser penetrates the cell vertically. Since all probes across the cell are excited, the overall fluorescence within the cell imposes a strong background on the single probes of interest, making the signal-to-noise ratio relatively low.

To improve the signal-to-noise ratio, total internal reflection fluorescence (TIRF) microscopy was invented to selectively excite fluorescent molecules close to the cover glass (<200 nm) (Axelrod 1981). TIRF is applied to visualize single molecule fluorescence near a surface (Khan* et al.* 2000; Lu* et al.* 2018; Sako* et al.* 2000; Vale* et al.* 1996; Wu* et al.* 2020) and especially to observe the diffusive motion of molecules on the cell membrane (Axelrod 2001). It is also used to track secretory granules in secretory processes (Reits and Neefjes 2001; Steyer and Almers 1999; Tsuboi* et al.* 2001; Zenisek* et al.* 2000).

Due to the observation depth, TIRF is limited to studying the cell membrane. To observe fluorescent molecules in cells, researchers further invented highly inclined and laminated optical sheet microscopy (HILO) (Tokunaga* et al.* 2008). The main difference between HILO and TIRF is the incident angle of the excitation light (Fig. 2B). In the HILO microscope, the excitation light no longer undergoes total reflection but penetrates and exits close to the interface, forming a thin layer of excitation light that illuminates the middle layer of the cell (Toomre and Bewersdorf 2010)**.**

### Data analysis

#### Trajectory

In SPT experiments, every probe is observed as a bright submicron spot described by the point spread function (PSF) due to the diffraction limit. We obtain an accurate position of the probe through Gaussian fitting. By linking the same particle’s different positions in consecutive images, particle trajectories are constructed (Fig. 2C). Both time and space information from the trajectories provide an opportunity to understand the characteristics of particle motion and further explore intracellular transport dynamics. Several algorithms are available (Cheezum* et al.* 2001; Chetverikov and Verestoy 1999; Sbalzarini and Koumoutsakos 2005; Tinevez* et al.* 2017; Vallotton* et al.* 2003) to help researchers obtain the trajectory of particles conveniently and efficiently.

#### Mean square displacement

To analyse the motion, the MSD of the trajectories is generally calculated:

$ < {\Delta r}^{2}\left(m\Delta t\right) > \;=\; < {(r\left(t+m\Delta t\right)-r(t))}^{2} > $

where *r*(*t*) is the displacement, Δ*t* is the time interval, and < ··· > is the average. Time-averaged MSD is:

$ < {{\Delta r}^{2}\left(m\Delta t\right) > }_{TA}=\frac{1}{M-m}{\sum }_{i\;=\;1}^{M-m}{\left[r\left(t+m\Delta t\right)-r\left(t\right)\right]}^{2} $

where *M* is the total time length of the trajectory. The ensemble-averaged MSD is:

$ < {{\Delta r}^{2}\left(m\Delta t\right) > }_{EA}=\frac{1}{N}{\sum }_{i\;=\;1}^{N}{\left[{r}_{i}\left(m\Delta t\right)-{r}_{i}\left(0\right)\right]}^{2} $

where *i* is the ID of each particle, and *N* is the total number of all particles. The two MSDs help us understand the motion of target particles, but their results are not always consistent. With increasing Δ*t*, the MSD tends to show an upward trend, which can be described by a remarkable power-law curve MSD ~Δ*t*^*α*^. The value of *α* depends on the motion type of the particle: *α* = 1 corresponds to Brownian motion, α < 1 refers to subdiffusion, and *α* > 1 refers to superdiffusion (Fig. 2D).

The diffusion rates are determined from the linear fitting of MSD:

$ < {\Delta r}^{2}\left(\Delta t\right) >\; =2dD\Delta t $

where *D* is the diffusion coefficient of particles, and *d* represents the dimensionality of space (Dupont* et al.* 2013; Ning* et al.* 2019).

#### Spatial distribution of intracellular diffusion

**Figure 3:**
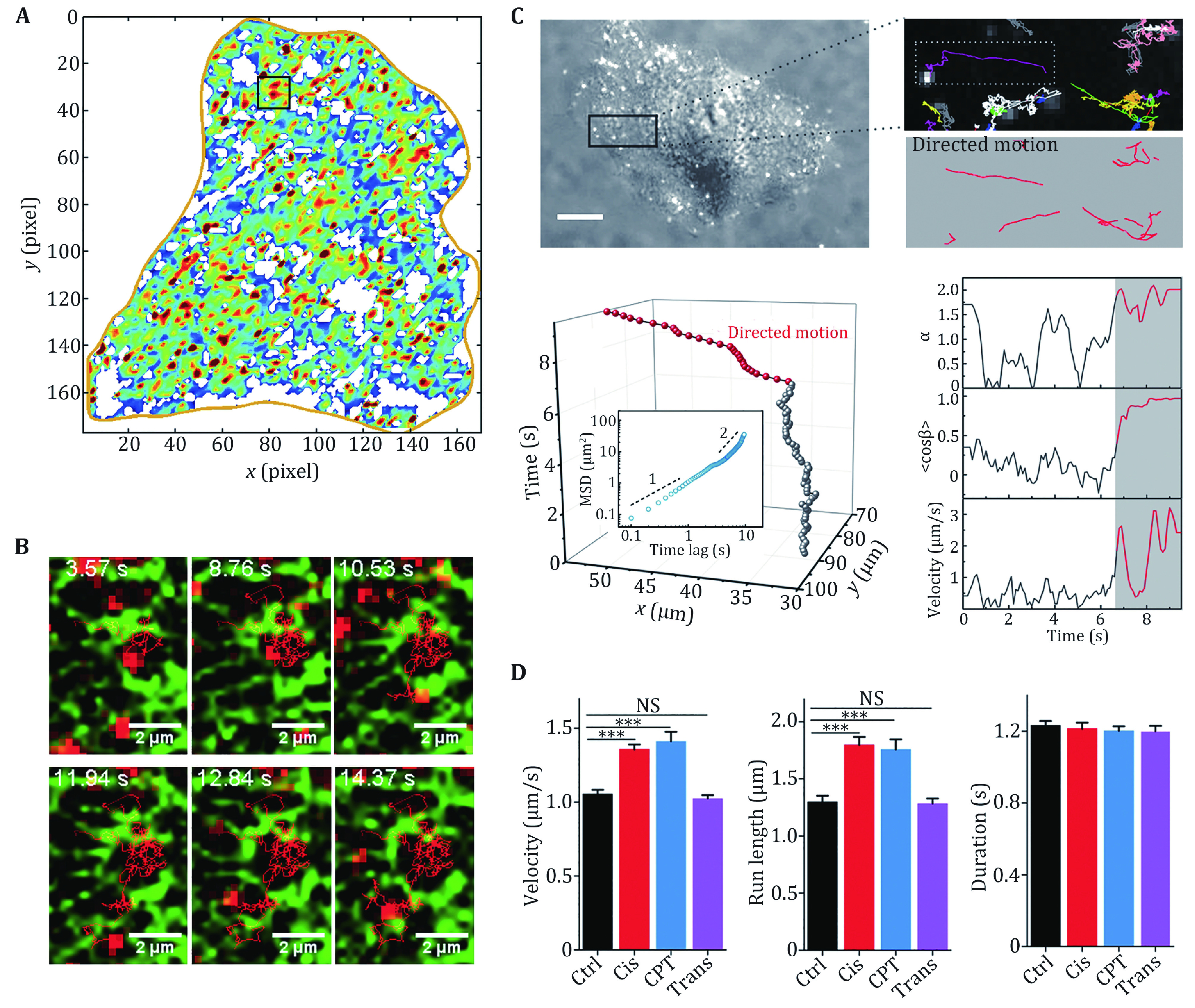
Diffusive motion and direction motion in living cells. **A** Diffusion map of a A549 human lung cancer cell, in which the local diffusion coefficients from 0 to 0.3 μm^2^/s are indicated by the colours from blue to red. **B** Image series of a QD trajectory (red) overlaid with GFP-ER (green). Reprinted with permission from American Chemical Society (Li *et al*. 2015b). **C** The trajectories of endocytic vesicles are divided into directed (red) and diffusive motion (grey). **D** Dynamical parameters of the velocity, run length and duration of the directed motion in early apoptotic cells. Reprinted with permission from National Academy of Sciences (Li *et al*. 2018a)

To describe the spatial heterogeneity of intracellular dynamics, one must focus on the trajectory within a specific spatial range. First, the cell is divided into different grids. Second, the segments of trajectories within a distance threshold to each grid node are chosen. Third, the ensemble-time-averaged MSD is calculated with the trajectory segments, and then the local diffusion rate is calculated by linear fitting of the first three points of the MSD. Fourth, with the diffusion rate at each grid node, the diffusion map of the cell is plotted. The parameters of grid size and distance threshold determine the final resolution of the diffusion map (Fig. 3A).

#### Extraction of the directed-motion segments

The typical trajectory of vesicle transport in cells consists of directed and diffusive motions. To extract the segments of directed motion, analysis of both the local MSD and directional persistence is applied to identify the local motion state of single particles in a time window. Compared with the MSD of a whole trajectory, the local MSD uses the particle positions around the time point of interest. Similarly, MSD can be fitted with MSD_local_ = *A*Δ*t*^*α*^, where *α* represents the nonlinear relationship of MSD with time. To further describe the direction information of particle motion, the local directional persistence $  < \mathit{cos}\beta  >     =\dfrac{1}{n}\displaystyle\sum \mathit{cos}{\beta }_{i} $ is applied, where *β*_*i*_ represents the change in angle between adjacent steps along the trajectory (Li* et al.* 2012a, 2018a). For a perfect unidirectional motion, <*cosβ*> = 1. The thresholds of *α* and *<cosβ>*can be set to judge whether the segments belong to the directional motion. Note that these parameters should be selected according to the experimental conditions. Further control experiments should be performed by eliminating all directed motion with cytoskeleton-disrupting drugs (Fig. 3C).

## SPT APPLICATIONS IN INTRACELLULAR TRANSPORT

Intracellular transport is the basis of many cellular functions, so it is important to understand the mechanisms of intracellular transport dynamics. In recent decades, due to its high spatial and temporal resolution, SPT has been widely employed to explore intracellular transport and proven to be a powerful and effective tool for measuring intracellular dynamics (Manzo and Garcia-Parajo 2015; Wang* et al.* 2021). SPT was first applied to study the biomolecule dynamics on the cell membrane, since the TIRF microscopy enables the single-molecule imaging on cell basal membranes with a high signal-to-noise ratio. Moreover, labelling of on-membrane biomolecules by fluorescence probes is relatively easy to achieve. Later on, with the improvements of HILO and 3D imaging techniques and the new generations of fluorescence probes, the SPT studies in the cytoplasm of living cells have been greatly promoted. Recently, the investigations are moving deeper into the nucleus. Despite that the complex intranuclear environment with dense chromatins has brought more difficulties to the labelling and tracking of single molecules, new methods of SPT are being developed. Here, although many advances have been made in SPT of intracellular transport, we can only introduce some representative studies in this review.

### Diffusion

#### Diffusion on the membrane

The membrane of living cells separates the internal and external environment of the cell. Cell membranes not only maintain the intracellular environment of stable cell metabolism but also regulate the exchange of substances between the intracellular and extracellular spaces. The cell membrane is mainly composed of fluid phospholipids and proteins that can move laterally (Jacobson* et al.* 2019). Moreover, the presence of the cytoskeleton beneath the membrane, lipid rafts, and other factors on the membrane may affect biomolecule motions.

SPT has contributed to important progress in studies of cell membrane dynamics. In 1993, Kusumi *et al*. used SPT to observe for the first time different movements of receptors on the cell membrane, including stationary mode, simple Brownian diffusion, directed diffusion, and confined diffusion (Kusumi* et al.* 1993). In 1994, Ghosh *et al*. observed anomalous diffusion of individual low-density lipoprotein receptors (LDL-Rs) on cell membranes (Ghosh and Webb 1994). Similar phenomena were further observed for other transmembrane receptors, including glycine receptor (GlyR) (Dahan* et al.* 2003), G-protein-coupled receptor (GPCR) (Calebiro* et al.* 2013), epidermal growth factor receptor (EGFR) (Chung* et al.* 2010), and acetylcholine receptors (AChRs) (He* et al.* 2016), which are attributed to molecular crowding and membrane heterogeneity (Kusumi* et al.* 2005; Owen* et al.* 2009; Saxton and Jacobson 1997). Moreover, Lippert *et al*. found that Wnt3A proteins bind to and diffuse on the plasma membrane of living cells without any receptor binding (Lippert* et al.* 2017).

The diffusive dynamics of lipids have also been studied by SPT. In 1996, Schmidt *et al*. applied SPT to analyse the movement of individual lipid molecules in an artificial membrane (Schmidt* et al.* 1996). It was further discovered by SPT that phospholipids undergo hop diffusion due to the compartmentalization of cell membranes (Fujiwara* et al.* 2002, 2016; Lagerholm* et al.* 2017).

It is worth mentioning that in addition to receptors and lipids, the diffusion of viruses and particles on membranes has been investigated by SPT. In 2005, Ewers *et al*. studied the lateral mobility of murine polyoma virus-like particles (VLPs) on cell membranes and artificial lipid bilayers using SPT (Ewers* et al.* 2005). Recently, a motion-pattern transition of single nanoparticles on the membrane was revealed (Ge* et al.* 2021).

#### Diffusion in the cytoplasm

With the development of SPT technology, it has been widely used to study the diffusion of intracellular molecules. Due to the viscoelastic properties of the cytoplasm and the presence of organelles, diffusion in the cytoplasm is quite complex. In 2013, Tabei *et al.* observed anomalous diffusion of insulin particles within the cell (Tabei* et al.* 2013). In 2015, Li *et al.* introduced a new method based on SPT to rapidly map intracellular diffusion, revealing heterogeneous and compartmentalized diffusion resulting from restriction of the endoplasmic reticulum (ER) (Fig. 3A, 3B) (Li* et al.* 2015b). In 2018, Zhao *et al.* further characterized the highly spatiotemporal heterogeneity dynamics of lysosomes in cells (Zhao* et al.* 2018). With the 3D SPT technique, it was recently found that intracellular diffusion is anisotropic quasi-2D rather than isotropic 3D in adherent cells (Chen 2020; Jiang* et al.* 2020). Han *et al*. used the diffusive dynamics of fluorescence beads to explore the intracellular dynamics between benign and malignant breast cancer cells (Han* et al.* 2020). The combination of SPT and superresolution microscopy enables the study of dynamics in organelles, such as the diffusion properties of proteins in mitochondria (Appelhans* et al.* 2012).

#### Diffusion in the nucleus

SPT also has applications in probing diffusive dynamics in the nucleus. In 2005, the Gratton lab tracked interphase chromatin dynamics using a two-photon excitation microscope, showing that chromatin in the nucleus undergoes confined diffusion and diffusional jumps (Levi* et al.* 2005). Moreover, individual telomeres in the nucleus of eukaryotic cells were found to exhibit anomalous diffusion on a short timescale and normal diffusion on a long timescale (Bronstein* et al.* 2009). The use of reflected light-sheet microscopy in combination with SPT improves the signal-to-noise ratio and enables the measurement of the diffusive dynamics of individual transcription factors in the nucleus (Gebhardt* et al.* 2013). Another work studied the diffusive dynamics of transcription factors and found the influence of nuclear architecture on gene regulation (Izeddin* et al.* 2014). In addition, the diffusion of microinjected viral ribonucleoprotein in the nucleus has been studied (Babcock* et al.* 2004).

### Directed motion

#### Vesicle trafficking

Endocytic receptor transport is a complex and dynamic process. The transport of endocytic vesicles contains directed motion driven by motor proteins along the cytoskeleton. Endocytic transport after internalization of the QD-ligand-receptor complex in real time has been revealed (Liang* et al.* 2007; Lidke* et al.* 2004, 2005; Rajan* et al.* 2008). Furthermore, the unidirectional and discontinuous transport of nerve growth factors in axons has been shown (Cui* et al.* 2007). In 2012, a study on EGFR endocytic trafficking found that paclitaxel altered the transport dynamics of endocytic vesicles by interfering with microtubule structures (Li* et al.* 2012b). In 2018, a study showed that the intracellular transport of endocytic vesicles is accelerated in the early stages of apoptosis due to increased intracellular ATP concentrations. Accelerated transport was demonstrated to be necessary for apoptotic progression (Li* et al.* 2018a) (Fig. 3C, 3D).

In addition to the translational motion, the rotation of single particles also provides important dynamic information. For example, the rotation of gold nanorods during endocytosis and subsequent intracellular transport was clearly shown (Chen* et al.* 2017; Xu* et al.* 2021). Moreover, the rotation of endosomes during neuronal axonal transport was observed by nanorods (Kaplan* et al.* 2018). In addition, endocytic transport of aptamer-drug conjugates was also characterized by SPT (Lv* et al.* 2019).

#### Viral infection

Viral infection is a complex process involving many steps and complex interactions with different subcellular structures (Cheng and Ghany 2020). After entering the cells by endocytosis, the intracellular transport of a single virus to its destinations is critical for viral duplication and other functions. SPT has significantly contributed to the mechanistic understanding of the viral infection process (Brandenburg and Zhuang 2007; Liu* et al.* 2020b). In 2003, the Zhuang laboratory tracked individual labelled influenza viruses in living cells and determined the internalization and endocytic transport of influenza viruses (Lakadamyali* et al.* 2003). The intracellular transport of a single virus mainly involves three processes: actin-dependent motion at the periphery of the cell, directed transport by dynein to the perinuclear region, and microtubule-dependent intermittent movement in the perinuclear region. SPT has also been used to elucidate the entry and internalization pathways of other viruses, such as poliovirus (Brandenburg* et al.* 2007).

#### Other directed transport

In addition to the directed transport of vesicles and viruses mentioned above, SPT has been used to uncover the directed transport of other intracellular components. In 2006, Courty *et al*. characterized the *in vivo* dynamics of individual kinesin motors labelled by QDs (Courty* et al.* 2006). In 2012, Coppola *et al*. used 3D-SPT to elucidate the dynamics of cationic liposome-DNA complexes in living cells and found that the complex mainly undergoes directed motion, in which microtubules play important roles (Coppola* et al.* 2012). In 2017, Katrukha *et al.* utilized QDs to analyse the role of cytoskeletal modulation in both passive and active intracellular transport (Katrukha* et al.* 2017). In 2018, a novel type of membraneless organelle named cytoophidium was found to show directed transport in fission yeasts, which is attributed to the myosin V with actin filaments (Li* et al.* 2018b).

## PERSPECTIVES

In this review, we first introduced the biological functions and physical mechanisms of intracellular transport and then briefly reviewed SPT technology and its applications in studying intracellular transport. In the future, more efforts should be made to elucidate the functional roles of intracellular transport dynamics, bridging the gap between physical behaviours and biological functions. As intracellular transport dynamics provide the physical basis for the transfer of matter, energy, and information, which is crucial for cellular activities and functions, cells can regulate their functions by alternating intracellular transport dynamics. For example, intracellular transport dynamics are tightly correlated with apoptotic progression (Li* et al.* 2018a). Moreover, the functions of diffusion in biochemical reactions and cellular activities remain to be elucidated (Brangwynne* et al.* 2009). Since SPT has been used to reveal the infection mechanisms of the influenza virus at the single-molecule level, it is expected that SPT will help us to understand COVID-19 infection in the future, which may contribute to designing specific drugs targeting the invasion and intracellular transport of COVID-19 (Ding* et al.* 2021; Shi 2020).

SPT has promoted the study of intracellular transport dynamics, but there are still some challenges. To date, most SPT studies on intracellular dynamics have been carried out at the cellular level *in vitro*; however, the environment in Petri dishes is quite different from that in real tissue (Li* et al.* 2021; Pampaloni* et al.* 2007), and the characteristics and functions of cells in 3D tissue remain to be investigated in the future (Han* et al.* 2020; Jiang* et al.* 2021). Therefore, SPT technology in 3D tissue imaging *in vivo* is of great significance and has profound prospects. To observe tissues, new 3D imaging techniques are needed. Lattice light-sheet microscopy (LLSM) technology is an effective method for observing deep tissue (Li* et al.* 2015a; Liu* et al.* 2018) and provides high spatial-temporal resolution for observing the subcellular dynamics within cells or tissues. In addition to microscope developments, new probes are also needed. Compared with visible light, a fluorescent probe with near-infrared (NIR) emission can achieve deeper penetration and better imaging quality, which is suited for live tissue imaging (Cai* et al.* 2019; Dai* et al.* 2021; Li* et al.* 2019; Smith* et al.* 2009). Single-walled carbon nanotubes (SWCNTs) have unique intrinsic fluorescence emission in the second NIR window (1000–1700 nm), which makes them candidate fluorescent probes for SPT in deep tissue (Bachilo* et al.* 2002; Hong* et al.* 2015; Welsher* et al.* 2009). In brain tissue, SWCNTs have been tracked to reveal the nanoscale organizational structure of the extracellular space (Godin* et al.* 2017). In addition, QDs in NIR emission are another promising probe for SPTs in deep tissue (Cassette* et al.* 2013; Liu* et al.* 2020a; Zhou* et al.* 2015). Recently, a new kind of QD emitting at 1600 nm allowed *in vivo* confocal 3D imaging of tumour vasculatures in mice at a depth of 1.2 mm (Zhang* et al.* 2018). Although SPT in real tissue is still challenging, it is believed that with the development of optical microscopy and NIR probes, SPT will extend the study of intracellular transport dynamics *in vivo*, with promising applications in biophysical studies and biomedical diagnosis.

## Conflict of interest

Ming-Li Zhang, Hui-Ying Ti, Peng-Ye Wang and Hui Li declare that they have no conflict of interest.
